# Union Meeting of the Connecticut Valley Dental Society, the New England Dental Society, and the Connecticut State Dental Association, at Springfield, Mass., October 23, 24, and 25, 1889

**Published:** 1890-08

**Authors:** 


### UNION MEETING OF THE CONNECTICUT VALLEY DENTAL SOCIETY, THE NEW ENGLAND DENTAL SOCIETY, AND THE CONNECTICUT STATE DENTAL ASSOCIATION, AT SPRINGFIELD, MASS., OCTOBER 23, 24, AND 25, 1889.[1]

(Continued from page 309.)

### DISCUSSION OF DR. ROBERTS'S PAPER.

*Dr. Parmele.*—I would like to ask what is an overdose, and what are the symptoms of an overdose?

*Dr. Roberts.*—According to my knowledge it is not necessary to be very careful, but a dose is from three to ten grains. In my illustration, I placed three grains in a glass of water, the patient did not get the benefit of the three grains, but perhaps two-thirds of it,—it stopped the hemorrhage. I had a case a week ago; the patient bled extensively all night; she had neglected her mouth, the tissues being in a diseased condition; the next morning I sent

---

[1] Reported for the INTERNATIONAL DENTAL JOURNAL by Geo. A. Maxfield D.D.S., Holyoke, Mass.

her a three-grain dose, and saw her husband at night, when he told me the hemorrhage had all stopped.

*Dr. Stowell.*—Does tannic acid have any local effect?

*Dr. Roberts.*—If I had a case where I wanted to use it locally, I should perhaps sprinkle it on. I used to have the patient take it with a spoon and pass it into the mouth.

*Dr. Bartholomew.*—As I understand it, tannic acid, when it has passed into the system, undergoes a change and is converted into gallic acid.

*Dr. W. H. Atkinson.*—There has not been enough observations made to enable us to thoroughly understand this subject. It is stated that this change does take place. I have had some cases where, when the gallic acid was administered directly, I had better success than when tannic acid was introduced. The danger is not in the circulation, but infiltration of the parts when it gets into the smaller vessels. The history of the case here cited, as I have heard it, is only observation without application, to determine the relation of the quantity given, the manner of administration, and the depleted condition of the patient, and whether the latter condition would seem to indicate that the hemorrhage was about to subside at the time the administration was made. The writer said it came from gum tissue; if this was the case I do not see why a local application would not answer, and be a very reasonable and successful way of treating it. In such a case I prefer to use the persulphate of iron. If it comes from the apex of the socket, it is caused by the mouth of the blood-vessels being caught in the socket and are thus unable to close on themselves. When this is the case the best thing to do is to take a burr and burr the end of the socket a little larger so as to allow the sides of the open-mouthed vessels to be free from the walls; they will then contract and the hemorrhage cease. I think it is a very good plan to continue giving tannic acid for several days after you have succeeded in arresting the hemorrhage, for there is a liability of its recurring when the slough comes off.

*Dr. Bartholomew.*—Would you prefer to give gallic instead of tannic acid?

*Dr. Atkinson.*—That would be a question. If I were so impressed at the time that that was the best thing to do I would do it. I have not enough science in my facts to distribute it around.

*Dr. Bartholomew.*—Tannic acid is a very old application, yet in many instances has failed to perform the work desired. Tannic acid, applied to a wound outside of the mouth to keep it from bleeding, is one of the best of antiseptic applications. Internally, I would give gallic acid directly, and you can administer that with perfect safety; take a teaspoonful in a glass of water and it will inevitably stop the hemorrhage. There is not a case on record that I know of where gallic acid has been administered but what it has performed the work. It coagulates the albumen, that when the action of the acid has reached the wound the thin blood has stopped. In ninety-nine cases in a hundred you need not use anything else but tannic acid if the bleeding be on the surface, but if you will administer the gallic acid, you will get an effect where tannic acid will fail entirely.

*Dr. Maxfield.*—Does the gallic acid cause a thickening of the blood throughout the body?

*Dr. Bartholomew.*—Yes, but it also slows down the action of the heart. It affects the nerves of the heart not injuriously and need not cause any alarm.

*Dr. Maxfield.*—Are you not running a good deal of risk by thickening of the blood?

*Dr. Bartholomew.*—I do not mean to say that the circulation of the whole system is coagulated, but the moment it strikes the atmosphere you get your thickening, and the force of the blood current is not so strong.

*Dr. Maxfield.*—I do not think we have enough data to support the assertions that have been made in regard to the action of gallic acid. What we want in these cases is an agent that will effect the vaso-motor nerves and cause them to contract on the blood-vessels. The agent that will best accomplish this is ergot, and I would advise its administration in troublesome cases of hemorrhage.

Subject passed.

### *Friday Morning Clinic.*

*President Bartholomew.*—I have arranged with Dr. Keefe, a practising physician of this city, to administer ether to the patient whom Dr. Curtis will operate upon this morning. Dr. Keefe has a method peculiar to himself. By his method the patient becomes etherized in a very short time and with very little trouble, and I know you will all be greatly interested in this method. I would like to have Dr. Maxfield keep the time and report as soon as the patient is ready for the operation.

*Dr. D. E. Keefe.*—Gentlemen, as far as I know, this method is original with myself, a full description of it was published in the *New York Medical Journal* of November 20, 1886, page 573. The great trouble in administering ether is at the beginning to give sufficient air with the ether so as not to strangle the patient, and then as soon as the patient gets accustomed to the ether to give only the ether vapor. I have overcome these objections. I simply use a large towel, folding it first four times lengthwise and placing a piece of paper the full length between the outside folds, then I roll it up into a cylinder, the size to be governed by the face of the patient; you want it large enough to cover the face, and not too large. Now we have a cylinder open at both ends, full size. I then saturate the lower part of the cylinder, that comes against the face, with the ether, then apply it to the face, and as the other end is wide open, the patient gets a great deal of air and also a great deal of ether vapor. A few breaths are sufficient for the patient to become accustomed to the ether. I then close the outer end of the cylinder, by bringing the sides together and holding them with my hand. The patient now only gets the ether vapor, and is very soon wholly etherized, and without any struggling or coughing. Of course, before so many, the patient is somewhat nervous, and the time may be a little longer than it would be if I were administering it in an office, where everything was quiet. The average time generally taken to bring the patient under ether by this method is only two and one-half minutes, and I only use one and seven-eighths ounces of ether. You can see what a small quantity of ether it takes. Now, chloroform I consider just as safe as any anæsthetic if you only know how to use it. You must remember it is very powerful. It holds the same relation to ether that a powerful locomotive does to the small toy engine, and to use it one should thoroughly understand this, and, realizing this, they will be able to have it wholly under their control. In administering chloroform, the average time is two and forty-three-sixtieths minutes, and the quantity three-eighths of an ounce; compare it with the amount of ether required, you see it takes only one-fifth as much. The best thing for dentists to use is, I think, what is called the one, two, three mixture; or the R. C. E. mixture, that is, one part alcohol, two parts chloroform, and three parts ether. With this mixture it takes only two and forty-sixtieths minutes and only about six-eighths of an ounce. I will now administer the ether to this patient and you can judge for yourself.

*Dr. Maxfield.*—I have taken the time and it has taken just three minutes and three-quarters.

Dr. G. L. Curtis, Syracuse, N. Y., then performed the operation, which consisted in removing the redundant condition of the mucous membrane of the upper lip, which allowed about three-quarters of an inch of mucous surface to appear, greatly disfiguring the face. The upper lip was also contracted by its cord, which was incised to allow the muscles of the lip to return to their normal condition. A section an inch and a half long and half an inch wide of the mucous membrane was dissected from either side of the centre of the lip, and the wound was united by means of sutures. The result was an entire success, there being no external wound or disfigurement whatever. At the close of this operation the union meeting was called to order, and a vote of thanks was given Dr. Curtis for his efforts in performing so successful an operation.

*President Bartholomew.*—The next thing on our programme is the discussion of the subject of pyorrhœa alveolaris, to be opened by Dr. M. L. Rhein, New York City.

*Dr. Rhein.*—More attention has been paid to this subject the past few years than ever before. I want to call attention to the necessity of first making a careful examination of your patient. We are all aware of the fact that there has been a great deal of discussion as to what constitutes pyorrhœa alveolaris. Many of the profession object to the use of this term, and it is a very poor term to signify all the trouble that comes under this head, yet I have not been able to find any other that will give a better definition, or one that will describe the variety of symptoms of this disease. That this is a local disease in which a thorough local treatment and cleanliness of the parts will cure is not any more true than of other diseases that have local manifestations. The discussions on this subject and the stand taken by so many men of eminence in our profession, and whose opinions are entitled to respect, is proof of the fact that this disease is not distinctive, but represents a variety of diseases which present themselves in the form of a discharge of pus around the necks of the teeth or from any of the alveolar portions. It is therefore necessary for us to make a very careful diagnosis of the trouble before we commence any treatment. It is this one fact that I wish to bring out most prominently in this discussion, and impress it on the minds of the dental profession. I do not believe the dental colleges pay sufficient attention to diagnosis. Heretofore the most of our treatment has been entirely mechanical, we have done only that which was in plain sight before us. When a man has a tooth to fill, he sees everything before him just as it is, and it is the same when he extracts teeth. As our profession has advanced we have taken up the treatment of diseases that embrace diseases of other portions of the body, so that it is absolutely necessary for us to be certain of what we are going to do before we undertake to treat such diseases. The idea that pyorrhœa alveolaris is caused only by uncleanliness we must set aside. The treatment of this disease in its true form is one so complicated with the rest of the human organism that no man ought to undertake the care or treatment of such a case unless he is certain of the condition of his patient. If I have made myself clear on this subject, and the gentlemen here present will place the proper value on it, I believe they will find it of great advantage to them. It is not necessary that we all should be good chemists, or that we should thoroughly understand pathology, for we here have an opportunity of calling in the skill of other specialists, and getting their advice regarding the physical or pathological condition of the patient whom we propose to treat. It is a good plan, therefore, for those who are not skilled in diagnosticating diseases, not to attempt the treatment of a case until after they have called in a specialist, one who can make a thorough examination of the whole body. Having a case presented to us that we call pyorrhœa alveolaris, the first thing necessary to decide is, whether it is the result of uncleanliness or not. Those who have seen many cases can generally decide this question. If we decide that the case is not the result of uncleanliness, the next step is to discover whether there is at the present time any general disability or any organ of the body in a pathological condition. Have a careful examination of the urine made. Sometimes you will make the discovery that some of the organs are in a diseased condition, of which previously there had not been the least suspicion. The mouth is the first part of the body that will show signs of any defects in the general circulation. Having established the fact that some of the organs are in a pathological condition will decide us in our treatment. Every known disease that afflicts the human organism often manifests itself as pyorrhœa alveolaris, and in many it is the first marked symptom. It only then requires a little common sense to be able to state to our patients what hopes we have of being able to restore to them the entire usefulness of their teeth. There is one form of this disease that puzzles us, and that is, where we do not find it as the result of uncleanliness and where we cannot at the time find any pathological disturbance. In these cases, if we will carefully question the patient and get a good history of their life, we will find that at some former time, perhaps only a few years, or maybe fifteen or twenty years back, they suffered from some severe disease, a disease that was very intense and which brought about this condition of the gums at that time. After the recovery of their health, and having paid no particular attention to the mouth, this trouble has gone on from bad to worse. The cleansing of the teeth with the brush has not helped matters any, as the tissues have remained in the same condition as left by the disease. These are cases that respond to the proper treatment with remarkable celerity, and are such that we all like to treat.

I do not propose going into any lengthy discussion as to the etiology of this disease. That we have obtained so many favorable results from following our own practical conclusions, we can speak confidently of what the treatment must be. The first thing necessary is to remove all forms of deposit that may be on the teeth; when you find a case where there are no deposits on the teeth, that is strong evidence that you will find in the patient a pathological condition of some organ. Having cleaned the roots of every extraneous matter, getting rid of any necrosed or carious condition, the teeth must then be held together in some manner so they cannot move. When it is only necessary to hold them together for a few weeks or months, as that length of time will bring about a healthy condition, then by tying the teeth together with dental floss, thoroughly waxed, and passing it across the teeth two or three times, will be sufficient. This is better than the finest wire you can command, as you can more easily get into the small spaces.

If the teeth will not stand well without being held together for a length of time,—one or two years,—it is poor policy to trifle with floss or wire, as the food will fill in the spaces and defeat all your attempts. In such cases it is much better to perform some permanent operation, and this must depend on the skill and ingenuity of the operator having it in charge. Keep all apparatus away from the necks of the teeth. In many cases we can put our retaining appliance on the grinding surfaces of the teeth. Our next care is to see that the pockets are in a thoroughly clean condition, for cleanliness is the secret of success in the treatment of pathological conditions existing in the gums. The treatment I have mostly relied on is to inject the pockets surrounding the roots of the teeth with a solution of equal parts of $H_2O_2$ and a one to five hundred solution of bichloride of mercury. The next thing is to get rid of any pathological change around the roots of the teeth, and to remove this you will have to use some powerful agent. One agent that is now quite generally used for this purpose is a combination of caustic potash and carbolic acids. Having removed this condition, it is necessary to see that your patients do not neglect to take care of the teeth. It will be necessary for them to use an antiseptic wash of some kind, and it must be used so that it will reach the pockets; it is necessary that these pockets be kept thoroughly clean, and a spray atomizer is excellent for this purpose.

*Dr. Brackett.*—I wish to express my appreciation of these remarks. I feel grateful to our friend Dr. Rhein that he has been able to point out what he has, and has shown us how much progress has been made. In so many instances, such special diseases as Bright's disease and uterine disease manifest themselves in the mouth. One great practical idea that has been so beautifully put to us is, that in the mouth we get the first indications of general manifestations of pathological conditions of different organs of the body, and, knowing this, we should be guided by these evidences of systemic changes.

*Dr. Curtis.*—This subject has interested me very much for the past five years, during which I have treated a large number of cases and tried to treat as practically as possible. I have followed Dr. Rhein very carefully in treating cases, and he certainly conducts cases from a scientific stand-point and with thoroughness. I have often referred my patients to him and he has been of great assistance to me. The fact that it is necessary to remove all foreign substances, also necrosed or carious portions that we may find, and to thoroughly support the teeth that they may form an anchorage afterwards, are the essential points that Dr. Rhein has brought up; then of the cleansing and preventing of formation of pathological conditions. I think we must go still further than local treatment. I find in a large percentage of cases that pyorrhœa alveolaris is a local symptom of a general disease; that the blood is very far below normal condition; the presence of oxide of lime and other crystals in almost all cases is also marked. These can be determined by tests of the urine, for this shows that by disintegration or lack of disintegration it fails to keep the blood toned to its natural functions. We find this condition in rheumatic and gouty patients, and very often in patients with this diathesis we find this disease manifested. To return the blood to its normal condition is very essential. I find in some cases an improvement of four per cent. in two or three weeks' time. I think we must combine the general treatment with our local treatment, for without this complete success cannot be hoped for. Many of the disturbances to the teeth and gums is from the filthy condition of the mouth. I cannot express this too strongly, but if taken in time, much of this can be avoided. But there are cases where there are manifestations of tartar,—I think some one has described it as serumal tartar,—that accumulates on or near the apex of the root. That is due to the abnormal condition of the blood.

*Dr. W. C. Barrett.*—I cannot trace all cases of pyorrhœa alveolaris as positively as has been done by Dr. Rhein, which, in my hands, assume most of the conditions already named. It is not always due to the condition of health, and on close examination you may find it is not due to kidney troubles at all. You find one case just as distinct as another and without any relation to the diseases mentioned. I will cite the case of two ladies. There was in one case not the slightest disturbance of the urine. There was no connection with any disease whatever; for both of the ladies were in perfect health. Their personal habits were as perfect as any one's could be, yet these cases have been under my hands for several years and are under my hands to-day, in age they are from thirty to thirty-five. One is a mother of several children and the other married several years and is without children ; both are standing above the ordinary height. They are women with good positions in life and are well nourished. Both of them are typical cases. There is absolutely no possible conditions of general disease. I have had examinations made of the urine, and everything is normal. Cases of pyorrhœa alveolaris are just as common in persons without the diseases mentioned as with. One says it is due to eating too much salt, because they never saw a person who had pyorrhœa alveolaris who did not use salt. Well, I never knew a person who did not use salt. Because we happen to find a person with kidney trouble and troubled with pyorrhœa alveolaris, we jump at the conclusion and say the former is the cause of the latter.

There are different phases of pyorrhœa alveolaris, because in one case there are pockets beside the root of the tooth and in another case there are no pockets. You cannot say one is pyorrhœa and the other is not. What is pyorrhœa alveolaris? It is necessary for us to go back and obtain a definition, that which shall distinguish it from everything else. My theory or your theory is not sufficient unless we can show what we claim. There is such a thing as caries of the alveolar wall aside from that which is called necrosis, a kind of breaking up. This may be pyorrhœa alveolaris, yet it is not necessarily so.

*Dr. Maxfield.*—A theory has recently been advanced that pyrrhœa alveolaris is a disease caused by the action of the pulp in the tooth, and the claim is made that this disease never occurs on pulpless teeth. I would like Dr. Barrett's opinion on this theory.

*Dr. Barrett.*—I have not thought much of pyorrhœa alveolaris in connection with pulpless teeth, but there is a serumal deposit on the roots of teeth which is not salivary calculus. It must be necessarily of sanguinous origin. In regard to pulpless teeth I do not think there will be this breaking down which is common of pyorrhœa alveolaris in teeth having living pulps, yet the pericementum may possibly form a deposit on the roots of the teeth. In some cases the serumal deposit is quite extensive. My impression is that pyorrhœa alveolaris is ordinarily connected with teeth having living pulps.

*Dr. Rhein.*—I cannot comprehend how Dr. Barrett has so poorly understood me. I have not claimed any particular trouble as being the cause of pyorrhœa alveolaris in attributing it to constitutional causes; but I state this fact, that we do get pyorrhœa alveolaris in many of the forms that we know it as resulting symptoms from almost any disorder that the system has or may have had. My continued experience makes me most positive on this subject. Men may get it from any trouble, and so may women. There have been a number of cases under my observation where the trouble has been nothing but the result of systemic disturbances.

*Dr. Barrett.*—Dr. Rhein says he finds it in all diseases, then attributes it to all diseases. He must find it in some one disease, and whenever he finds it in some one particular case, then he has established a point that is connected with that particular disease. He must establish this connection, and he has not done so yet.

*Dr. Rhein.*—The point I make is, pyorrhœa alveolaris is a local manifestation of malnutrition of the parts, and we get malnutrition around the surfaces of the teeth before it manifests itself in any other part of the body. If there is any lack of stimulant in the circulation, it is going to be felt in the mouth first before it is felt in any of the extremities or larger organs of the body; and the conditions there are lack of nutrition, and not receiving this, they cannot remain in the condition which they have in state of health. What is more natural than that we should have retrograde action due to any disorder with the entire system in common. He asks for further proof, and to give this we must have some years yet to establish these things. I have been working over a year to establish these facts. I may find myself mistaken; if I am, I will be perfectly willing to admit it.

*Dr. W. H. Atkinson.*—It is certainly in my observation of this disease of all that comes to me I attribute to loss of natural power in the elements of the parts involved; that it may be present in connection with many other unhealthy conditions goes without saying. Some of the most clearly-defined cases I have seen have been in persons otherwise in perfect health. I would say that it is known, and sufficiently within our grasp to learn much from it. What is the nature of the disease? Never necrosis. I never have seen any cases that could be at all legitimately termed necrosis. How does mercury expend its force in the mouth? The teeth get loose by the melting away around the necks of the integument, *and it goes down one side of the root.* It was these cases that influenced me to be thorough in cleaning out the parts to get good results. Chemistry is what we want to understand, pathological chemistry. Retrograde metamorphosis has two methods: one is disposure and slow nourishing until parts become irritated; the other is where normal nutrition is overbalanced so as to give us an abnormal blood supply.

Subject passed.

(To be continued.)

